# Measuring Availability, Prices and Affordability of Ischaemic Heart Disease Medicines in Bangi, Selangor, Malaysia

**DOI:** 10.21315/mjms2019.26.5.10

**Published:** 2019-11-04

**Authors:** Huay Woon You, Nur Syamilah Athirah Tajuddin, Yusuf Al-Mubin Shaharin Anwar

**Affiliations:** Pusat GENIUS@Pintar Negara, Universiti Kebangsaan Malaysia, Bangi, Selangor, Malaysia

**Keywords:** ischaemic heart disease, medicine prices, affordability, availability, private sector

## Abstract

**Background:**

This study is aimed to analyse the availability, prices and affordability of medicines for ischaemic heart disease (IHD) in Bangi, Selangor, Malaysia.

**Methods:**

A quantitative research was carried out using the methodology developed by the World Health Organization and Health Action International (WHO/HAI). The prices were compared with international reference prices (IRPs) to obtain a median price ratio. The daily wage of the lowest paid unskilled government worker was used as the standard of the affordability for the medicines. In this study, ten medicines of the IHD were included. The data were collected from 10 private medicine outlets for both originator brand (OB) and lowest-priced generic brand (LPG) in Bangi, Selangor.

**Results:**

From the results, the mean availability of OB and LPG were 30% and 42%, respectively. Final patient prices for LPG and OB were about 10.77 and 24.09 times their IRPs, respectively. Medicines that consumes more than a day’s wage are considered unaffordable. Almost half of the IHD medications cost more than one day’s wage. For example, the lowest paid unskilled government worker would need 1.4 days’ wage for captopril, while 1.2 days’ wage to purchase enalapril for LPG. Meanwhile, for OB, the costs rise to 3.4 days’ wage for amlodipine and 3.3 days’ wage for simvastatin.

**Conclusion:**

The findings of this study emphasise the need of focusing and financing, particularly in the private sector, on making chronic disease medicines accessible. This requires multi-faceted interventions, as well as the review of policies and regulations.

## Introduction

In Malaysia, ischaemic heart disease (IHD) remained as the principal causes of death in 2017 (1). IHD is caused by the fatty material referred to as fatty tissue build up within the walls of arteries over time. This is often referred to as coronary-artery disease. If this build up happens within the arteries that provide the heart with blood, it is referred to as coronary cardiovascular disease. Eventually, the arteries might become slender, as the build up hinders deliverance of sufficient oxygen-rich blood to the heart, thus causing angina, which is the pain and discomfort causes by the build-up (2).

Access to a basic medical treatment is a right for human. Hence, providing access to medical care with affordable cost to patients and society has become a key health policy globally (3). However, up to 90% of the population in developing countries purchase medicines through out-of-pocket payments, making medicines the largest family expenditure item after food. As a result, medicines, particularly those with higher costs, may be unaffordable for large sections of the global population and are a major burden on government budgets (4).

In the past, numerous studies on medicine prices and affordability have been conducted, including IHD medicines. A study reported that four IHD medicines (aspirin, β blockers, angiotensin-converting enzyme inhibitors (ACE), and statins) were potentially unaffordable for 0%–14% of households in high-income countries, 25% of upper middle-income countries, 33% of lower middle-income countries, 60% of low-income countries and 59% households in India. Moreover, patients are less likely to use all four medicines if it is not affordable to them (5).

In addition, a study performed a review of coronary artery disease in Malaysia, where it showed that Malaysians are having Acute Cardiovascular Syndrome (ACS) at a younger age compared to other developed countries, with a mean age of between 55.9 and 59.1 years compared to mean ages of between 63.4 and 68 years in most developed countries. On top of that, this study stated that the evidence continues to show that coronary artery disease is the nation’s major cause of fatality and morbidity, requiring greater efforts to educate the public to change dietary habits and lifestyles, and to increase awareness of healthy living such as regular exercise (6).

It is known that patients with IHD need to take their medicines regularly to reduce the risk of getting a heart attack which could lead to a sudden death (7). Mourik et al. (8) summarised the availability, price and affordability of cardiovascular medicines from 36 countries using the World Health Organization/Health Action International (WHO/HAI) data. From the findings, there was great variability for all measures. This indicates that continuing research in this area is necessary (8). In light of this, the aim of this study is to investigate the availability, prices and affordability of IHD medicines, specifically in Bangi, Selangor, Malaysia.

The remainder of this article is structured as follows: Section 2 presents the methodology implemented in this study, Section 3 elaborates the results and discussion and finally some concluding remarks are drawn in Section 4.

## Materials and Methods

This study follows the WHO/HAI methodology (9, 10). The minimum numbers of private medicine outlets are five for a survey area. Nevertheless, the WHO/HAI also suggested that increasing the numbers of medicine outlets above the minimum recommended numbers will increase the survey’s accuracy (9). This survey was conducted in Bandar Baru Bangi, Selangor Darul Ehsan, Malaysia. The selected private sector samples should be within three hours’ travel from the main public health facility. Hence, a total of 10 private medicine outlets have been selected and surveyed.

Surveys were performed according to a standardised protocol with results double-entered into a uniform workbook for data analysis. In each survey, data on availability and patient prices were collected in a sample of private medicine outlets. Availability is expressed as the percentage of facilities where the medicine is found on the day of the data collection. Only formulations of the same strength and dosage form were included. Price is expressed as a price per unit (e.g. tablet, dose) and converted to a median price ration (MPR) by dividing the median local price by an international reference price (IRP). The IRP is obtained from the Management Sciences for Health (MSH) price indicator guide (11). The IRPs are the medians of recent procurement or tender prices offered by not-for-profit and revenue-driven suppliers to developing countries for multi-source products (12). Price ratios are not calculated when the medicine was present in less than four outlets. Whereas, treatment affordability is estimated by calculating the number of day’s wage the lowest unskilled government worker needs to purchase one month’s supply of medicine according to a standard treatment regimen. The size of the variation between 25th and 75th percentile was taken as a measure of price variation between all facilities surveyed (13).

Among 10 medicines for IHD included in the survey, three belong to the core list medicines suggested by WHO/HAI for international comparison, and seven were added as supplementary medicines (9). These were the most-surveyed medicines. The supplementary list was prepared based on the IHD needs as determined by a pilot community survey, while accounting other factors, such as the medicine utilisation patterns. All the medicines studied are listed in Table 1. For each medicine, data on patient prices and availability were collected for both originator brand (OB) and lowest priced-generic (LPG).

## Results

### Availability

Table 2 displays the percentage availability for the 10 IHD medicines. From Table 2, availabilities of IHD medicines ranging from 0% to 90% among the private medicine outlets in Bangi, Selangor. The mean availability of OB and LPG was 30% and 42%, respectively in the private sector. Based on National Essential Medicine List (NEML) from (14), the availability of the survey medicines listed on the NEML (excluding furosemide and lovastatin) was found with 33.75% and 42.50% for the OB and LPG, respectively.

For the OB products, amlodipine had the highest availability (i.e. 90%), while captopril and hydrochlorothiazide were not available in the private medicine outlets samples. On the contrary, atenolol and simvastatin had the highest availability for the LPG with availability percentage of 70%. Meanwhile, captopril and nifedipine retard recorded the lowest availability for LPG, i.e. 20%. Among the 10 medicines, seven LPGs had a higher availability than OB products, except amlodipine and nifedipine retard. In addition, the simvastatin had a similar percentage availability for the LPG and OB. Moreover, the mean availability of generic medicines were more than the originator brands by 12% (i.e. 42%–30% = 12%). Regardless of the brand of the medicine, analysis of all medicines availability showed that amlodipine has the highest mean availability which was 75% while captopril has the lowest mean availability among the private sector which was just 10%.

Table 3 lists the availability of individual medicines in the private medicine outlets. Only eight OBs and 10 LPGs were found in the private sector with only 36% availability as a whole, based on Table 2, which is quite low for IHD patients as this is the main killer disease in Malaysia.

### Median Price Ratios and Medicine Prices Variation

Table 4 summarises the MPR for the 10 IHD medicines, which ranged from 11.99 for simvastatin to 46.64 for lovastatin for OB and from 2.02 for simvastatin to 31.10 for lovastatin for LPG. Across the IHD medicines, the highest MPR was found in lovastatin (OB) with 46.64, while the lowest MPR, 2.02, was found in simvastatin (LPG).

Overall, based on Table 4, the private sector procured 10 generics at 10.77 times their IRPs and eight OBs at 24.0 times their IRPs. The MPRs of eight OBs–amlodipine [17.99], atenolol [22.90], enalapril [14.33], furosemide [40.84], lovastatin [46.64], nifedipine retard [15.58], propranolol [22.46] and simvastatin [11.99] were more than 10 times their IRPs. Ten LPGs were procured at higher prices than the IRPs; six medicines were more than five times the reference price, including captopril [10.76], enalapril [20.91], furosemide [11.72], lovastatin [31.10], hydrochlorothiazide [8.87] and propranolol [10.37].

Further analysis of 10 medicines for which OBs and generically equivalent products showed that in the private sector, OB cost 63.63% more, on average, than their generic equivalents. Thus, patients pay substantially more for originator brand medicines when lower-cost generics are unavailable.

Low variation across all the medicines was noted for both OB and LPG medicines in the private sector. Based on Table 4, the overall price variation for OBs was 1.74 while for LPGs was 1.49. Hence, price variation for OBs was higher than LPGs in this study by 14.4%.

### Affordability

The affordability of treatments for IHD medicines are given in Table 5. In general, OB products were less affordable than the LPG equivalents in the private sector. The affordability of LPGs in the private sector had reasonable affordability (with standard treatment costing a day’s wage or less) for nearly all LPGs medicines. The exception for this were captopril and enalapril. The most affordable standard treatment was hydrochlorothiazide with 0.2 days’ wage to purchase one month of treatment. When OBs are prescribed and dispensed in the private sector, some treatment costs much higher than the LPGs. For example, treating IHD with OB of amlodipine required 3.4 days’ wage while LPG of amlodipine only required 0.8 days’ wage.

**Figure 1:**
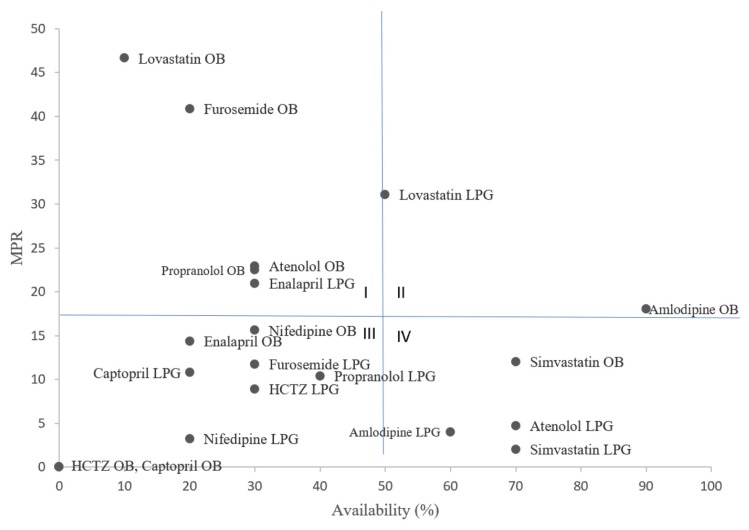
Medicine availability and MPR for OBs and LPGs

Figure 1 summarises the availability and price of IHD medicines in the 10 private medicine outlets. The percentage availability for each medicine is depicted on the *x*-axis, while the *y*-axis shows the value of MPR. The figure can be divided to four quadrants. Quadrant IV contains medicines with low MPR and high availability, for example simvastatin (OB) which has 70% availability and a MPR of 11.99. Quadrant I contains medicines with high MPR and low accessibility. Patients face high financial burden and trouble in getting them. For example, lovastatin (OB) was available in only 10% of surveyed private sectors and cost over 46.64 times the IRP. Hence, lovastatin (OB), furosemide (OB), propranolol (OB), atenolol (OB), and enalapril (LPG) making IHD treatment potentially challenging in Bangi, Selangor.

Notably, some generics are easily accessed at low cost in the private sectors, such as amlodipine (supplementary), atenolol (global core) and simvastatin (global core). This can be concluded that Bangi, Malaysia is in the right path in maintaining the usage of global core medicines for IHD as two out of three surveyed global core medicines have high availability in the study area.

## Discussion

Availability of medicines is important for patients’ access to treatment. The average availability of medicines in Bangi, Malaysia was 36% in the private sector. However, availabilities of over 50% were observed in some medicines (OB and/or LPG). This study also found that the availability of LPG was higher than originators in the private sector which reflects the country’s Generic Medicines Policy. Since Malaysian National Medicines Policy (MNMP) was endorsed by the Malaysian Cabinet in 2006, generic medicines have been widely used in the public sector, which influence the private sector too. This is one of the government’s initiative to promote the use of generics in the community (15).

The highest price variation goes to simvastatin (OB) with 4.04 and propranolol HCl (LPG) with 3.00 (see Table 4). This shows that the variation of prices for simvastatin and propranolol HCl were high across different medicine outlets, which means that the gap between the prices varied distinctively. Aside from that, the rest of the medicines have low price variation which were near to 1.00. Thus, the price variation for the IHD medicines was generally low according to this study. This result reflected that medicine outlets in Bangi follow the market price recommended by the supplier. The patient prices of originators were fairly stable across private premises as there was only a single brand for each medicine in the market. Previous works in Malaysia demonstrated that prices of originators indirectly serve as a cap to generics as the latter are still relatively affordable even after significant mark-ups (16). Nevertheless, the Good Pharmaceutical Trade Practice (GPTP) guideline was published by the (15) to promote standard price and bonus scheme to all distribution channels and health care providers. This rule received additional support from the Malaysia Competition Commission (MyCC), though it is not legitimately authoritative. Therefore, adherence is poor as execution by pharmaceutical organisations is purely voluntary. Hence, combined with competition among various generic brands for each medicine in the market, this study may clarify the patient price variations across generics observed in this study.

According to (9), treatments that needs less than a day’s wage are considered affordable. In relation to this, some medicines in this study were affordable, such as amlodipine (LPG), atenolol (LPG), enalapril (OB), furosemide (LPG), hydrochlorothiazide (LPG), lovastatin (LPG), nifedipine retard (LPG), propranolol HCl (OB and LPG), and simvastatin (LPG) based on Table 5. Generally, the LPG were more affordable. This can be supported with the results of this study which shows that only two OBs were affordable (enalapril, 0.8 days’ and propranolol HCl, 0.8 days’), while two LPGs were not affordable (captopril, 1.4 days’ and enalapril 1.2 days’). This indicates that OBs were less affordable for chronic disease medicines in the private sector. These results are in line with previous studies, see (17–19), to name a few. Although the low-income population may utilise the public health care, there is still a proportion of the population that visit the private sector and may not be able to afford continuous treatment (13).

## Conclusion

This analysis demonstrates that the average availability of IHD medicines was 36% in private medicine outlets, in which the LPGs were higher than OB products. For the affordability of treatments, the medicines are classified as affordable when it requires less than a day’s wage of the lowest paid unskilled government worker. From the findings, nearly all the LPG medicines are affordable. However, despite the affordability of generic medicines, certain innovator medicines are unaffordable (amlodipine, atenolol, furosemide, lovastatin, nifedipine retard, simvastatin). Further, prices of medicines which procured much higher than the IRPs should be reviewed, for example, Lovastatin (OB and LPG) and Furosemide (OB). As with patient prices, the availability varied among the IHD medicines.

Looking at these results, future research works can be extended to include the public sector and compare the availability, prices and affordability between private and public sectors in Bangi, Selangor, Malaysia. Besides, future studies are also recommended for other chronic disease medicines, such as pneumonia.

## Figures and Tables

**Table 1:** List of IHD medicines

No.	Medicines	Type
1.	Amlodipine 10 mg	Supplementary
2.	Enalapril 20 mg	Supplementary
3.	Furosemide 40 mg	Supplementary
4.	Hydrochlorothiazide 25 mg	Supplementary
5.	Lovastatin 20 mg	Supplementary
6.	Nifedipine retard 10 mg	Supplementary
7.	Propranolol HCl 40 mg	Supplementary
8.	Atenolol 50 mg	Global Core
9.	Simvastatin 20 mg	Global Core
10.	Captopril 25 mg	Global Core

**Table 2:** Percentage availability for the 10 IHD medicines

Medicines	Brand	Overall		OB	LPG
Amlodipine	OB	90	75	30	42
	LPG	60			
Atenolol	OB	30	50		
	LPG	70			
Captopril	OB	0	10		
	LPG	20			
Enalapril	OB	20	25		
	LPG	30			
Furosemide	OB	20	25		
	LPG	30			
Hydrochlorothiazide	OB	0	15		
	LPG	30			
Lovastatin	OB	10	30		
	LPG	50			
Nifedipine retard	OB	30	25		
	LPG	20			
Propranolol HCl	OB	30	35		
	LPG	40			
Simvastatin	OB	70	70		
	LPG	70			
Total				36	

**Table 3:** Availability of medicines in the private medicine outlets

Availability	Originator brand	Lowest-priced generic
Medicines not found in any outlets	Captopril, Hydrochlorothiazide	-
Medicines found in less than 25% of outlets	Enalapril, Furosemide, Lovastatin	Captopril, Nifedipine retard
Medicines found in 25% to 50% of outlets	Atenolol, Nifedipine retard, Propranolol HCl	Enalapril, Furosemide, Hydrochlorothiazide, Lovastatin, Propranolol HCl
Medicines found in 50% to 75% of outlets	Simvastatin	Amlodipine, Atenolol, Simvastatin
Medicines found in over 75% of outlets	Amlodipine	-

**Table 4:** Median price ratio (MPR) of OBs and LPGs in the private sector

Medicine name	Brand	Median price ratio	Price variation (Q75/Q25)	Minimum price	Maximum price
Amlodipine	Brand	17.99	1.33	6.43	34.70
	Lowest price	4.02	1.41	2.89	6.36
Atenolol	Brand	22.90	1.99	6.70	36.03
	Lowest price	4.69	1.32	3.75	21.43
Captopril	Brand	-	-	-	-
	Lowest price	10.76	1.30	7.95	13.57
Enalapril	Brand	14.33	1.60	7.74	20.91
	Lowest price	20.91	1.13	16.73	21.54
Furosemide	Brand	40.84	1.40	27.363	54.33
	Lowest price	11.72	1.09	9.77	11.72
Hydrochlorothiazide	Brand	-	-	-	-
	Lowest price	8.87	1.39	8.32	14.97
Lovastatin	Brand	46.64	1.00	46.64	46.64
	Lowest price	31.10	1.30	21.77	67.38
Nifedipine retard	Brand	15.58	1.39	9.34	19.17
	Lowest price	3.23	1.77	1.44	5.03
Propranolol HCl	Brand	22.46	1.17	17.28	24.19
	Lowest price	10.37	3.00	3.46	31.10
Simvastatin	Brand	11.99	4.04	2.02	20.65
	Lowest price	2.02	1.14	1.57	3.59

**Table 5:** Treatment affordability for 10 IHD medicines

Medicines	Medicine strength (mg)	Dosage form	Treatment duration (days)	Brand	Days’ wage*
Amlodipine	10	Cap/tab	30	OB	3.4
				LPG	0.8
Atenolol	50	Cap/tab	30	OB	2.1
				LPG	0.4
Captopril	25	Cap/tab	30	OB	-
				LPG	1.4
Enalapril	20	Cap/tab	30	OB	0.8
				LPG	1.2
Furosemide	40	Cap/tab	30	OB	1.3
				LPG	0.4
Hydrochlorothiazide (HCTZ)	25	Cap/tab	30	OB	-
				LPG	0.2
Lovastatin	20	Cap/tab	30	OB	1.1
				LPG	0.7
Nifedipine retard	10	Cap/tab	30	OB	2.7
				LPG	0.6
Propranolol HCl	40	Cap/tab	30	OB	0.8
				LPG	0.4
Simvastatin	20	Cap/tab	30	OB	3.3
				LPG	0.6
